# On the Neutral Azotized Materials of Organization

**Published:** 1843-07

**Authors:** MM. Dumas


### PART FOURTH.

### Medical Intelligence.

### On the Neutral Azotized Materials of Organization. By MM. Dumas and Cahours.

Many of our readers may be aware that the correctness of several of Liebig's views is impugned by the eminent French chemist Dumas; and that, in regard to some others, a claim for priority is set up by the latter. Into these questions we shall not at present enter, but shall confine ourselves to a statement of the most important results which have been obtained by an extensive series of analyses lately concluded by the authors of this paper. These analyses were made upon a larger scale than most of those which have preceded them, in order that the proportion of azote, which is not only the most important item in the whole, but the one most difficult of determination, might be ascertained with the greatest exactness.

1. *Albumen.* These experiments appear to prove satisfactorily, that albumen possesses the same composition in all animals, and *à fortiori* in all the liquids of the same animal. Vegetable albumen does not differ from animal albumen in elementary composition; but it is not united with free soda, as it ordinarily is in the animal body. There is a much closer agreement between the several analyses made by MM. Dumas and Cahours, of albumen obtained from a variety of sources, than there is between the three analyses of M. Scherer (which have been taken as the standard by Liebig and others) of the albumen of the serum of blood only. This, to our minds, is good evidence of the accuracy of the methods employed. The average composition of five different kinds of animal albumen is as follows:

| | | |
|---|---|---|
| Carbon . . . . . . . . | 53·42 | |
| Hydrogen . . . . . . . | 7·19 | |
| Azote . . . . . . . . | 15·74 | |
| Oxygen, sulphur, and phosphorus . | 23·65 | = 100·00 |

In no instance was a departure from this average indicated, to the amount of more than ·1 or at most ·2 per cent.,—a degree of conformity extremely remarkable.

2. *Casein.* This substance, when drawn from the milk of herbivorous animals, presents the same composition in each case, and properties nearly identical. The milk of the human female, who approaches the carnivorous quadruped in her habits of life, furnishes a casein which, whilst possessing a composition identical with that of the casein of herbivorous quadrupeds, exhibits peculiar properties, which may perhaps become the groundwork of future distinctions. In some abnormal conditions of human blood, there exists a substance which seems to resemble casein, both in composition and properties, but the authors have not succeeded in detecting it in the blood of animals during lactation. The flour of the cerealia includes a substance which seems analogous to casein, presenting the same elementary composition, and its most important properties. All the varieties of *casein* possess the same elementary composition as *albumen,* and these two substances are certainly *isomeric.*

3. *Legumin.* A remarkable and very distinct substance has been found in the kernel of the almond and other similar fruits, as well as in the leguminous seeds, which has been regarded by Proust, Vogel, Liebig, and others, as identical with animal casein. According to our authors, however, this substance bears a nearer resemblance to gelatin in regard to composition, though it completely differs from it in properties. It deserves peculiar attention, on account of its abundance in the alimentary materials just mentioned, and the part which it seems to take in the nourishment of the animal body. For this substance, when dissolved in dilute muriatic acid, communicates to it exactly the same properties with albumen; whence it may be inferred that, under the influence of the gastric juice, it will furnish the same soluble products as albumen itself. It appears probable that this substance consists of a mixture or combination of albumen or casein with some other product; but as this mixture appears to be made in constant proportions, there is no inconvenience in retaining for the present the term *legumin,* which was proposed by M. Braconnot for the substance extracted from peas and beans. Legumin, then, in a physiological view, consists of a substance analogous either to albumen or to casein, but mixed and combined with another body richer in azote, which modifies its most important properties. Without doubt, the nutritive power of *legumes* is determined in great degree by the proportion of legumin which they contain; but it would be premature to consider this substance as performing a part identical with albumen or casein. A portion of the elements of legumin are combined in a peculiar state, which probably renders them less adapted for aliment than those which are united in the exact proportions which constitute albumen and casein.

4. *Fibrin.* It seems to be a very definite and positive conclusion from analyses of MM. Dumas and Cahours, that fibrin, instead of being identical with albumen, (as recently maintained by Liebig and his school,) in the proportion of the four elements—oxygen, hydrogen, carbon, and azote, differs from it very sensibly. The following are the results of these analyses, which certainly present a very remarkable accordance.

| | Blood of | | | | | | | |
|---|---|---|---|---|---|---|---|---|
| | Sheep. | Calf. | Ox. | Horse. | Dog. | Dog A. | Dog B. | Man. |
| Carbon | 52·8 | 52·5 | 52·7 | 52·67 | 52·74 | 52·77 | 52·57 | 52·78 |
| Hydrogen | 7·0 | 7·0 | 7·0 | 7·0 | 6·92 | 6·95 | 7·07 | 6·96 |
| Azote | 16·5 | 16·5 | 16·6 | 16·63 | 16·72 | 16·51 | 16·55 | 16·78 |
| Oxygen, &c. | 23·7 | 24·0 | 23·7 | 23·70 | 23·62 | 23·77 | 23·81 | 23·48 |

The dog A had been fed during two months and a half upon meat alone, whilst the dog B had been fed during the same time on bread only.

Upon comparing the average of these analyses with that already given for albumen, it is evident that the proportion of carbon is about ·7 per cent. less in fibrin than in albumen, whilst the proportion of azote is from ·8 to ·9 per cent. greater. The difference is so striking and constant, that it cannot be attributed to accidental causes. A tolerably just idea of the elementary composition of fibrin might be formed, by regarding it as a combination of casein or albumen, and ammonia. This idea would seem, at first sight, to derive confirmation from the fact, that when fibrin (previously well washed) is boiled for a long time in water, the liquid which is distilled over contains ammonia, whilst the insoluble residue has the composition of ammonia. But as fibrin may be dissolved in a solution of caustic potass, without losing its excess of azote, it is not probable that this azote exists in it in the state of ammonia. There can be little doubt that the prolonged boiling produces an actual change of composition. Our readers will bear in mind the fact supposed to be ascertained by M. Bouchardat, that after fibrin has been long boiled, a substance which he regards as analogous to gelatin is found in the water. (See Brit. and For. Med. Rev., vol. XV. p. 229.) It might hence be thought that the excess of azote, and the smaller proportion of carbon, in fibrin, is due to an admixture of gelatin with the albuminous matter of which it chiefly consists, since this difference in elementary composition is characteristic of gelatin. But the fibrin, whilst thus setting free the (so-called) gelatinous matter, *also* disengages ammonia; and, according to MM. Dumas and Cahours, this matter is very unlike true gelatin, either in its composition or its properties; for it does not form a jelly on cooling, and it is precipitated by nitric acid; and it contains a smaller proportion both of carbon and azote, and a considerably greater amount of oxygen. Hence they consider it not improbable that, in the prolonged action of boiling water upon fibrin, there is an actual decomposition of the water and of the animal substance; and that the oxygen of the water fixes itself in the latter, whilst the hydrogen sets free some of its azote in the form of ammonia.

From the general properties of fibrin, it seems certain that this substance contains a large proportion of a product identical with albumen or casein, which it yields to dilute muriatic acid; and that, consequently, it undergoes the same action with the gastric fluid with these substances. Hence, as an alimentary material, it may be classed with them; but as a product of animal life it must be regarded in a very different light, since its physiological properties are far more distinct from those of albumen, than are its chemical ones.

The question naturally arises, whether there is a *vegetable fibrin* equally distinct from vegetable albumen. This also appears to have been resolved by MM. Dumas and Cahours, the former of whom indicated the existence of such a substance in the year 1839. It may be obtained from wheat flour, by using cold or tepid water in the analysis; and its composition *then* resembles that of animal fibrin. But if it be treated with boiling water (as was the case in the analyses of Dr. Jones) it is converted into a substance having the composition and properties of albumen.

5. Independently of these four principal products, albumen, casein, legumin, and fibrin, there are two others which approach them in their mode of action with dilute muriatic acid, so as evidently to appertain to the same group, although their properties seem at first sight altogether distinct. These are *glutin* and *vitellin.* The former is one of the substances extracted from gluten, and is distinguished by its solubility in boiling alcohol, from which it can only be separated by evaporation. The ultimate composition of this substance appears to be the same with that of albumen and casein; but it has certain special properties, and appears to be the matter chiefly concerned in the pannary fermentation. Its full history, however, is yet to be developed. The *vitellin,* which constitutes the albuminous matter of the *yelk* of egg, nearly approaches albumen and casein in its properties; but it differs considerably in composition, a much larger proportion of oxygen being present in it.

The authors agree with Liebig in thinking that these *albuminoid* substances are the only materials from which most of the animal tissues are formed; and that neither the non-azotized substances, nor gelatin, can have any share in their production. They proceed to examine the ultimate destiny of these substances, and to calculate the amount of heat which will be produced by their *combustion,*—a combustion of which they estimate the following as the results:

| | | | | |
|---|---|---|---|---|
| 1 Atom of Albumen, consisting of C. 48, H. 37, Az. 12, O. 15, together with 100 additional atoms of Oxygen ................ | is converted into | C. 6, | H. 12, Az. 12, O. 6, | or 3 Urea. |
| | | C. 42, | O. 84, | or 42 Carb. Acid. |
| | | | H. 25, O. 25, | or 25 Water. |

All that they say on this subject exhibits the tendency to regard the animal body as a sort of machine, that is, (like a steam-engine,) to gain a certain amount of power by the combustion of a given quantity of fuel, on which we have elsewhere remarked, as fundamentally erroneous.

*Annales de Chemie,* Dec. 1842.

